# The influence of the career calling on the employees’ creative deviance

**DOI:** 10.3389/fpsyg.2022.1069140

**Published:** 2022-12-15

**Authors:** Xiwei Liu, Yunbao Xu

**Affiliations:** School of Management, Hunan Institute of Engineering, Xiangtan, Hunan, China

**Keywords:** career calling, ethical leadership, creativity, creative deviance, innovation

## Abstract

As creative deviance can improve organizational innovation ability and competitiveness effectively, scholars have recently paid much attention to this innovative manner. This paper examines the mechanism between career calling and creative deviance based on 304 surveyed samples *via* a cross-temporal questionnaire. The results show that employees’ career calling exerts a significant positive impact on their creative deviance, and employee creativity plays a mediating role in this relationship. Furthermore, ethical leadership positively moderates the relationship between career calling and employee creativity as well as the relationship between career calling and creative deviance.

## Introduction

Creative deviance implies the behavior that employees adhere to their innovation ideas when their innovation plan is inconsistent with the organizational innovation strategy or being refused by their leaders (Mainemelis, 2010; Lin et al., 2016). The existing literature finds that firms which regard absolute unity as core value tend to show poor innovation ability, while firms which can tolerate employees’ deviant behaviors prone to be more creative and competitive (Emeth, 1997; Plucker and Gorman, 1999). Due to the rapid deterioration of the external environment faced by enterprises, the creative deviance becomes an innovative way to help enterprises gain competitiveness (Liu X. Q. et al., 2021). Nevertheless, some enterprises may ostensibly support diversified innovation while suppress or ignore creative deviance in the workplace since it deviates from organizational expectations (Ford, 1996; Criscuolo et al., 2014). As a result, many employees will give up such innovative manner. Therefore, how to motivate employees’ creative deviance behaviors has attracted much attention in recent years (Lin et al., 2016).

**Figure 1:**
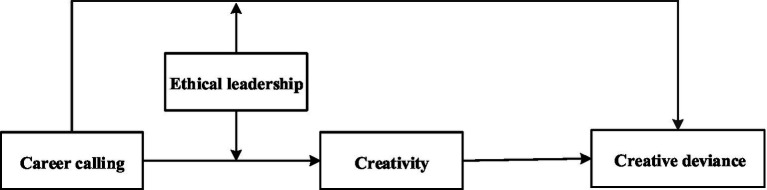
Theoretical model of research.

In the process of innovation, organizations are more likely to choose innovative ideas that can quickly transform innovative results into market products because of considerations such as norms, benefits and leaders’ preferences. Instead, those innovative proposals with relatively high cost and long investment cycle may be rejected (Mainemelis, 2010). Intriguingly, while most employees may abandon their innovation ideas, a few employees tend to hide their innovation activities from leaders or disobey orders to continue their proposed innovation activities, i.e., creative deviance (Globocnik and Salomo, 2015; Lin et al., 2016). Nevertheless, the studies on why these employees can persist in creative deviance are still sparse. With the rapid development of China’s economy, employees’ pursuit of economic rewards is less fanatical than before, thus many scholars call for researches on promoting employees’ positive behaviors from the perspective of career calling (Liu Z. et al., 2021; Chen et al., 2022). Career calling implies the willingness of individuals to engage in certain jobs to realize their value and meet their self-realization needs (Duffy et al., 2012a). Since career calling guide individuals to perform their job duties aligned with social values and promote common progress of the organization and society (French and Domene, 2010), we guess that employees with higher career calling may be more responsible for their work and are more likely to insist on their innovation ideas even without superiors’ supports. Moreover, since creative deviance is essentially an innovative behavior which can be affected by individual innovation ability, career calling may stimulate more innovative ideas through increasing individual work engagement and mutual communication (Tenzer and Yang, 2020). Hence, whether employee creativity can mediate the relationship between career calling and creative deviance could be explored. Furthermore, the literature documents that employees’ individual values and work attitudes can be influenced by leadership styles (Badrinarayanan et al., 2019; Shakeel et al., 2020). Since ethical leadership which can influence individuals’ normative behaviors through role modeling, scholars call for further empirical researches on its effect on “abnormal” behaviors of employees (Velez and Neves, 2018; Shakeel et al., 2020). We echo this call by taking ethical leadership as a boundary condition. In the light of above, our theoretical model is shown in Figure 1.

Our research makes several theoretical contributions to literature. First, we explored why employees engage in creative deviance. Previous studies focused on environmental, organizational or leadership factors (Lin et al., 2016; Sarpong et al., 2018; Petrou et al., 2020; Liu X. Q. et al., 2021), while overlooked the effect of employees’ personal traits. Taking employees’ career calling as antecedents, we discussed its positive effect on creative deviance, and thereby enrich the research on the antecedents of creative deviance and provide a new perspective for motivating the employees’ innovation behavior. Second, we elaborated how career calling affects employees’ creative deviance. The findings show that career calling will promote employees’ creativity and help them to break conventions, question leaders’ decisions and reorganize knowledge effectively, thus exhibit more creative deviance (Yao et al., 2020a; Liu Z. et al., 2021). Herein, this study could enrich the literature concerning the antecedents of creativity and complement an understanding of the influence path between career calling and creative deviance. Finally, we elucidated when will career calling exert stronger effect on employees’ creative deviance. Existing studies have emphasized the influence of leadership style on employees’ innovative behaviors (Oluwafemi et al., 2020; Yao et al., 2020b; Nazir et al., 2021), we chose ethical leadership as a boundary condition and found its moderating role in the relationship between career calling and creative deviance as well as the relationship between career calling and creativity. Thus, we responded to the calls of previous scholars and broadened the literature on career calling and its effect.

## Theory and hypothesis

### Career calling and creative deviance

Calling is a theological and religious concept rooted in the western cultural context. Originally, it implies a condition in which an individual is called by God to act as a priesthood (Xie et al., 2016; Ahn et al., 2017; Kim et al., 2017). After development, it evolves into career calling and signifies the sense of mission at work (Xie et al., 2016; Ahn et al., 2017; Kim et al., 2017). To date, although several studies have discussed the definition of career calling, they have not reached a consensus yet (Shimizu et al., 2019). We summarizes the literature and propose three characteristics of career calling: First, career calling can be obtained from the inner belief, highlighting the individual’s perception and attitude toward works (Peng et al., 2019; Zhang and Jiang, 2020). Second, career calling has a strong sense of purpose and meaning, emphasizing the realization of self-worth (Duffy et al., 2013; Tian et al., 2018). Third, individuals with strong career calling have strong prosocial motivation and will take the initiative to help others, underscoring the promotion of social progress (Dik and Duffy, 2009; Duffy et al., 2012a; Praskova et al., 2015). Therefore, we suppose that career calling implies the belief that an individual is willing to engage in a certain job in accordance with a value advocated by the society, so as to realize their self-value and promote the common progress of both individuals and society (Dik and Duffy, 2009; Dik et al., 2012). Hence, stimulated by altruistic tendencies and prosocial motivations, individuals with high-level career calling tend to show strong work enthusiasm and responsibility consciousness (Xie et al., 2016; Ahn et al., 2017; Kim et al., 2017), and are willing to share social responsibilities for the organization to make their lives more meaningful (Dik et al., 2012; Duffy et al., 2012b).

The literature on creative deviance highlights that when employees adhere to their innovation ideas in spite of the leaders’ opposition, they may bring more benefits to the organization (Mainemelis, 2010; Lin et al., 2016). Drawing on the self-consistency theory, individuals are driven to unify their cognition and behaviors. If cognitive differences exist, individuals will exhibit the behavior in line with their values (Lin et al., 2018; Wu et al., 2018). Thus, we speculate that employees with career calling will align with the organizational values and insist on innovative behaviors which can promote the organizational interests (i.e., creative deviance). More specifically, first, employees with career calling are more willing to dedicate themselves to the organization (Dik and Duffy, 2009; Dik et al., 2012; Lobene and Meade, 2013; Liu Z. et al., 2021). If employees believe that their innovative idea can benefit the organization, they are more likely to adhere to implement this innovative idea even when their ideas are rejected by the organization. For instance, they may hide their innovation activities from their leaders or disobey orders to continue their proposed innovation activities (i.e., exhibit creative deviance). Second, employees with stronger career calling have a higher level of work passion (Liu Z. et al., 2021). They tend to be more diligent and are more willing to meet various challenges in the work (Lin et al., 2016; Tenzer and Yang, 2019). In this case, creative deviance is more likely to occur (Hall and Chandler, 2005; Ng and Yam, 2019). Finally, employees with higher level career calling may have stronger prosocial motivation. Insisting on their innovative ideas not only can benefit the organization, but also can promote the social progress (Hagmaier and Abele, 2012). For example, new products developed through creative deviance can achieve self-worth and benefit the society. Therefore, we propose the following hypothesis:

*Hypothesis 1*: Career calling positively correlates with employees’ creative deviance.

### The mediating effect of creativity

Employee creativity implies the ability to generate novel ideas or develop marketable products by knowledge recombination (Hennessey and Amabile, 2010). Employees with higher creativity are more capable to identify innovation opportunities, and thereby improve the innovation performance of the organization (Biraglia and Kadile, 2017). Prior studies offer considerable evidence on the relationship between employee creativity and external factors such as leadership style (Rego et al., 2014; Wang et al., 2019), while the role played by the internal variable is largely overlooked by the academic research (Hirschi, 2012; Thuan and Thanh, 2019). In fact, as a type of personal trait, employee creativity might be affected by personal inner cognition and belief more easily.

Considering such internal stimulus, we suggest that career calling can promote employees’ creativity. First, as a kind of inner belief, career calling has been regarded as a critical factor to increase career happiness (Duffy et al., 2018, 2019). On the one side, career calling can enhance employees’ job satisfaction and job engagement (Hirschi, 2012), which can boost their internal work motivations and positive work attitude (e.g., work passion), and thereby facilitate the generation of innovative ideas (Duffy et al., 2015; Liu Z. et al., 2021). On the other side, Hirschi (2012) finds that career calling enables employees to invest more energy in works and focus more on work efficiency and work achievement. As the literature noted, employees’ work motivation can exert great impacts on the creative deviance (Dik and Shimizu, 2019). Second, career calling provides strong work motivation and internal driving force for employees to achieve self-worth. According to self-determination theory, internal driving force of employees is one of the motivations of self-determination (Deci and Ryan, 1985; Ryan and Deci, 2006). Since career calling provides employees with sufficient driving forces, working in self-determination mode can boost them to create new ideas. Similarly, based on the motivation-job matching theory, Amabile (1993) illustrates that when individuals are genuinely devoted to work and not distracted by external factors, they can come up creative ideas more easily. Moreover, employees with higher career calling are more willing to accept challenging work, during which they can generate more novel ideas and thereby enhance their creativity (Duffy et al., 2016). Third, employees with career calling tend to be altruistic and prosocial, making them more favorable among organizational members. In this way, employees can communicate with colleges more frequently and facilitate knowledge sharing between them, thereby improving employees’ creativity (Duffy et al., 2012a, 2013; Yao et al., 2020a).

The literature has proven that the more creative an employee is, the stronger divergent thinking and critical thinking he/she will have (Hennessey and Amabile, 2010). Divergent thinking, a way of thinking that breaks routine and pursues innovation, emphasizes recombination of knowledge and unique thinking (Chaubey et al., 2019; Chaubey and Sahoo, 2019). Critical thinking emphasizes active thinking, introspection and questioning, demonstrating that individuals make reasonable criticism through repeated argumentation and questioning (Tan et al., 2017). We argue that both divergent thinking and critical thinking could increase the possibility of employees’ creative deviance for the following reasons. First, creative deviance is an innovative behavior that breaks organizational rules and regulations, which embodies the core of divergent thinking: breaking conventions and pursuing innovation. Since individuals’ behaviors consist with their cognitive thinking, employees are more likely to exhibit creative deviance behavior if they have divergent thinking (Gino and Wiltermuth, 2014). Likewise, critical thinking enables employees to question leaders’ decisions about innovative ideas and provide supports for ideas through own experiments, which will surely promote the creative deviance of employees (Mainemelis, 2010; Lin et al., 2016; Tan et al., 2017). Furthermore, employees with higher creativity have higher innovation self-efficacy and intrinsic motivation, which exert positive effect on their creative deviance (Lin et al., 2016; Tenzer and Yang, 2019). Therefore, we propose the following hypothesis:

*Hypothesis 2*: Employee creativity mediates the relationship between career calling and creative deviance.

### The moderating effect of ethical leadership

Ethical leadership implies a leadership style that leaders manage employees by role modeling in accordance with organizational norms (Treviño et al., 2003; Men et al., 2020). It conveys three connotations: First, the leader obey social ethics strictly to protect the interests of both subordinates and the organization. Second, the leader shows respect for employees and is willing to accept their suggestions. Third, the leader plays a role model and influences employees’ behavior positively (Velez and Neves, 2018; Badrinarayanan et al., 2019). Besides setting clear ethical standards, ethical leadership will supervise employees to implement standards. Indeed, ethical leadership is portrayed as fair, upstanding and careful, which shares several similarities to transformational leadership and authentic leadership, nonetheless, it put more emphasis on following ethical standards and caring for subordinates (Lee et al., 2019; Shakeel et al., 2020). Drawing on social learning theory, individual behaviors (especially complex behaviors) are affected by innate factors and acquired environment. Hence, besides learning through innate response results (direct experience), individuals can also adjust their behaviors *via* observing behaviors of excellent organizational members (indirect experience; Nahrgang et al., 2009; Lee et al., 2019). For employees, their leader is undoubtedly the first choice to learn and observe behaviors (Treviño et al., 2003; Zhang and Jiang, 2020).

Under a high-level of ethical leadership, leaders will take the initiative to care for employees. In this situation, employees will be more active in improving their innovation performance in return (Ozsungur, 2019a; Ye et al., 2022). Driven by such motivation, employees with career calling will pay more attention to the importance of creativity in the work which can benefit the organization, and strive to enhance their creativity by coming up with creative ideas (Walumbwa et al., 2011; Lee et al., 2019; Men et al., 2020; Shakeel et al., 2020). Meanwhile, ethical leadership can create a friendly work environment, under which employees with high-level career calling tend to communicate with others due to their altruistic and pro-social motivations (e.g., knowledge sharing), and thereby create brilliant ideas through exchanging thoughts (Treviño et al., 2003; Walumbwa et al., 2011; Chen and Hou, 2016; Goswami and Agrawal, 2022). In addition, compared with other leadership styles, ethical leadership are more tolerant to employees, making employees with career calling are more likely to exhibit creative deviance for the organizational benefits (because leaders may not punish the employee even if they know that the employee hides their innovation activities). Furthermore, ethical leadership tend to give employees higher degree of autonomy and uphold the legitimacy of rejected ideas if employees comply with regulations and moral norms. In this scenario, career calling will motivate employees to pursue benefits for the organization, which can not only satisfy their self-realization needs but also help leaders increase organizational competitiveness (Dik and Duffy, 2009; Duffy et al., 2012b, 2013).

In contrast, under a low-level of ethical leadership, demonstration effect of the leader will be diminished, making employees can hardly learn from leaders. Moreover, leaders take less care of employees, which in turn decreases employees’ appreciation. As a result, employees’ achievement motivation, pursuit of self-worth as well as pro-sociality will decrease (Walumbwa et al., 2011; Lee et al., 2019; Shakeel et al., 2020), causing them pay less attention to innovative ideas and thus injure their creativity (O’Keefe et al., 2018; Ozsungur, 2019a). Furthermore, working in a less tolerant environment will make employees always worry about whether the leader will blame their creative deviance behaviors or encroach upon their innovation achievements (Liu et al., 2012; O’Keefe et al., 2018; Ozsungur, 2019b). In addition, fears of being scolded by the leader will make employees reluctant to put extra efforts into creative deviance (Mainemelis, 2010; Lin et al., 2016; Lee et al., 2019; Shakeel et al., 2020). Accordingly, we propose the following hypotheses:

*Hypothesis 3*: Ethical leadership moderates the positive impact of career calling on employee creativity. In other words, the positive effect of career calling on employee creativity is more pronounced when the ethical leadership is higher.

*Hypothesis 4*: Ethical leadership moderates the positive impact of career calling on employees’ creative deviance. In other words, the positive effect of career calling on employees’ creative deviance is more pronounced when the ethical leadership is higher.

## Materials and methods

### Sample and procedure

The samples are full-time research and development (R&D) employees of manufacturing companies in the Chinese cities of Shanghai, Hangzhou, Changsha and Wuhan (mainly engaged in machinery production and R&D, the company scale more than 800 people, and established more than 10 years). To avoid serious common method variance, we collected data in two time points. At Time 1, participants filled out their basic information and scales of career calling and ethical leadership. A month later, at Time 2, they filled out scales of employee creativity and creative deviance. All the scales were self-reported by employees. To make the samples’ data close to the real value, we explained to all participants that the questionnaires are anonymous before distributing the questionnaire, enabling them to fill in the questionnaire truthfully. During the questionnaire distribution process, we waited near them to address their queries about questionnaires. After the questionnaire was filled out, it was immediately coded, retrieved and sealed. A total of 400 questionnaires were issued.

All questionnaires were filled voluntarily, after eliminating the invalid questionnaires with too many missing values, regular answers, two investigation mismatches or obvious disqualification, we collected 346 questionnaires a Time 1 and 304 at Time 2, with the overall response rate of 76.00%. Among them, males account for 54.93% and females 45.07%; The mean age was 33.442 (SD = 6.801). In terms of educational background, 41.78% of the participants were undergraduates or below, 58.22% were postgraduates or above. The average length of service is 7.059 (SD = 5.843).

### Measures

This study adopted 5-point Likert scale (ranging from 1 “strongly disagree” to 5 “strongly agree”) and had undergone a strict translation-back process. The scale sources were as follows.

#### Career calling

The CVQ questionnaire of Dik and Duffy (2009), calling scale of Dobrow and Tosti-Kharas (2011) and the calling scale of Chinese people developed by Zhang et al. (2015) were used for measure career calling, including “my work can help me find the meaning of life,” etc., with a total of 12 items. In this study, Cronbach’s *α* = 0.806.

#### Ethical leadership

A nine-item scale developed by Zheng et al. (2011) was used to measure ethical leadership, and sample items includes “listens to what employees have to say.” In this study, Cronbach’s *α* = 0.757.

#### Employee creativity

We used a four-item developed by Farmer et al. (2003) for measurement, and sample item is “seeks new ideas and ways to solve problems.” In this study, Cronbach’s *α* = 0.872.

#### Creative deviance

We used the scale developed by Lin et al. (2016) in the Chinese context, including “I continued to improve some of the new ideas, although they did not receive my supervisor’s approval,” etc., with a total of 9 items. In this study, Cronbach’s *α* = 0.796.

#### Control variables

We refer to the literature on employees’ creative deviance (Lin et al., 2016; Gutworth and Hunter, 2017; Shukla and Kark, 2020), and take gender (0 = male, 1 = female), age, education background (0 = bachelor degree or below, 1 = graduate degree or above) and working years (since employees entered the company) as the control variables for study.

### Validity testing

We use AMOS 22.0 to conduct confirmatory factor analysis for core variables (i.e., career calling, ethical leadership, creativity and creative deviance), aiming to test the discriminative validity of scales. Table 1 shows that the four-factor model has the best fitting effect and is evidently better than other candidate factor models, demonstrating that our scale has good discriminant validity.

**Table 1:** Confirmatory factor analysis results.

Models	χ^2^/df	△χ^2^	IFI	TLI	CFI	RMSEA
Four – factor model^d^	2.143	—	0.931	0.925	0.928	0.064
Three – factor model^c^	3.721	815.943^***^	0.893	0.901	0.908	0.094
Two – factor model^b^	6.848	2433.790^***^	0.813	0.811	0.814	0.116
Single – factor model^a^	10.214	4177.494^***^	0.712	0.684	0.691	0.163

^a^Career calling, Ethical leadership, Creativity, Creative deviance.

^b^Career calling + Ethical leadership, Creativity, Creative deviance.

^c^Career calling + Ethical leadership, Creativity + Creative deviance.

^d^Career calling + Ethical leadership + Creativity + Creative deviance. + represents the combination of the two before and after factors into a factor, ^***^p < 0.001.

Although we collect questionnaires at two stages to reduce the common method variation, it is difficult to avoid since the research variables in this study are all from the self-evaluation of employees. Therefore, we used the Harman single factor test, which shows that the first factor only explains 25.056% of the total variance. Besides, the fitting effect of the single-factor model is not ideal (χ^2^/df = 10.214, IFI = 0.712, TLI = 0.684, CFI = 0.691, RSMEA = 0.163), which illustrates that common method variation do not exert a serious impact on the research results.

## Results

### Descriptive statistics

Table 2 presents descriptive statistics and correlation analysis. Employee career calling significantly positively correlates with creativity (*r* = 0.303, *p* < 0.01) and creative deviance (*r* = 0.356, *p* < 0.01). Moreover, there is a significant positive correlation between employee creativity and creative deviance (*r* = 0.375, *p* < 0.01). The results provide preliminary support for our research hypotheses.

**Table 2:** Mean, standard deviation and correlation coefficient matrix.

	1	2	3	4	5	6	7	8
1. Sex	N/A							
2. Age	−0.062	N/A						
3. Education	−0.050	0.069	N/A					
4. Working years	−0.036	0.475^**^	−0.118^*^	N/A				
5. Career calling	0.006	0.013	0.001	0.038	***0.806***			
6. Ethical leadership	0.049	−0.033	0.034	−0.003	0.575^**^	***0.757***		
7. Creativity	−0.058	0.032	−0.079	−0.008	0.303^**^	0.462^**^	***0.872***	
8. Creative deviance	−0.056	0.104	−0.038	0.057	0.356^**^	0.257^**^	0.375^**^	***0.796***
Mean	0.450	33.442	0.582	7.059	3.087	3.229	3.594	3.301
SD	0.498	6.801	0.494	5.843	0.413	0.405	0.746	0.409

*n* = 304, ^**^p < 0.01, ^*^p < 0.05, two-tail test, the bold and diagonal is the value of Cronbach’s α.

### Hypothesis testing

#### Main effect test

We first conduct multicollinearity test on all variables. The results demonstrate that all variance inflation factors are <2 and the tolerance is >0.100, indicating that there is no serious collinearity problem in the sample data. Furthermore, we use SPSS 23.0 software to conduct hierarchical regression analysis, and the results are shown in Table 3. According to M5, career calling significantly positively correlates with creative deviance (*b* = 0.352, *p* < 0.001), thereby supporting Hypothesis 1.

**Table 3:** Regression analysis results.

	Creativity			Creative deviance			
	M1	M2	M3	M4	M5	M6	M7
**Control variables**							
Sex	−0.092	−0.095	−0.124	−0.043	−0.045	−0.030	−0.028
Age	0.006	0.007	0.009	0.006	0.006	0.005	0.005
Education	−0.139	−0.142^+^	−0.172^*^	−0.039	−0.041	−0.019	−0.016
Working years	−0.006	−0.008	−0.008	0.005	−0.003	0.002	0.004
**Independent variable**							
Career calling		0.452^***^	0.306^**^		0.352^***^	0.265^***^	0.285^***^
**Mediating variable**							
Creativity						0.157^**^	0.154^**^
**Moderating variable**							
Ethical leadership			0.236^**^				0.146^**^
**Interaction items**							
Career calling × Ethical leadership			0.116^+^				0.113^*^
*R* ^2^	0.013	0.106	0.283	0.016	0.142	0.215	0.294
△*R*^2^		0.093^***^	0.177^***^		0.126^***^	0.073^***^	0.079^***^

*n* = 304, ^***^p < 0.001, ^**^p < 0.01, ^*^p < 0.05, ^+^*p* < 0.1.

#### Mediating effect test

Table 3 shows that employees’ career calling significantly positively correlates with their creativity (M2, *b* = 0.452, *p* < 0.001) and employee creativity significantly positively correlates with creative deviance (M6, *b* = 0.157, *p* < 0.01). To further validate the mediating effect of employee creativity, we use the PROCESS plug-in of SPSS 23.0 software to conduct the Bootstrapping mediation effect test, and the results are displayed in Table 4. The Bia-corrected 95% confidence interval of the indirect effect of career calling on creative deviance is [0.048, 0.142], excluding 0. Therefore, the mediating effect of creativity exists and H2 is supported.

**Table 4:** The mediating effect test.

Variables	Bootstrapping			
	Bia-Corrected 95%CI		Percentile 95%CI	
	Lower	Upper	Lower	Upper
Indirect effect				
Career calling → Creativity → Creative deviance	0.048	0.142	0.047	0.142
Direct effect				
Career calling → Creativity → Creative deviance	0.159	0.369	0.159	0.369

*n* = 304, Bootstrapping for 5,000 random sampling.

#### Moderating effect test

**Figure 2:**
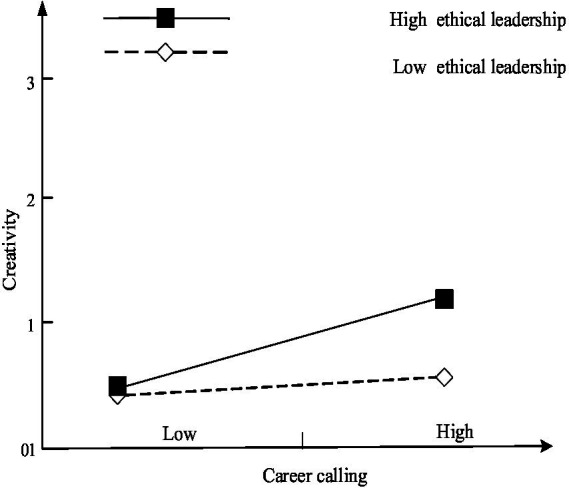
Result of simple slope test (1).

**Figure 3:**
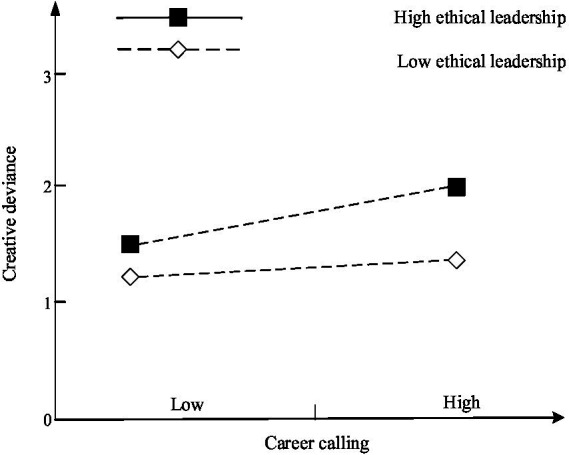
Result of simple slope test (2).

Table 3 demonstrates that the interaction item between career calling and ethical leadership exerts a significant positive effect on employee creativity (M3, *b* = 0.116, *p* < 0.1) and creative deviance (M7, *b* = 0.113, *p* < 0.05). The simple slope test shows that career calling exerts significant positive influence on creativity (*b* = 0.353, *p* < 0.1) and creative deviance (*b* = 0.331, *p* < 0.1) under high level ethical leadership (Mean + 1 SD). While career calling exerts no significant impact on creativity (*b* = 0.259, *p* = 0.258) and creative deviance (*b* = 0.239, *p* = 0.296) under low level ethical leadership (Mean-1 SD). Hence, career calling exerts more pronounced positive impact on employee creativity and creative deviance under the high leadership, and H3 is supported. Figures 2, 3 show the simple slope test, respectively.

## Discussion

While creative deviance may reduce the efficiency of organizational management, it can enhance organization’s innovation ability and competitiveness (Emeth, 1997; Plucker and Gorman, 1999; Backstrom and Bengtsson, 2019; Rangus and Cerne, 2019). Many scholars find that enterprises which can tolerate employees’ creative deviance are more stable and competitive (Ford, 1996; Criscuolo et al., 2014). Therefore, encourage employees to exhibit creative deviance properly can help organizations acquire new ideas and improve performance. Drawing on self-consistency theory and social learning theory, this paper examines the mechanism between career calling and creative deviance based on 304 surveyed samples *via* a cross-temporal questionnaire. We find that employee career calling exerts a significant positive impact on both creativity and creative deviance, and employee creativity mediates the relationship between career calling and creative deviance. We further find that ethical leadership positively moderate the relationship between career calling and creativity as well as the relationship between career calling and creative deviance. More specifically, the positive influence of career calling on employees’ creativity and creative deviance will be more pronounced under a high level of ethical leadership.

### Theoretical implications

This paper makes several contributions to the literature. First, we elucidate the positive effect of career calling on employees’ creative deviance. Although previous researches have explained the effect of employee creative deviance, its antecedent variables and influencing mechanism remained largely unexplored. Since some studies highlight that creative deviance can be affected by internal factors such as personal characteristics (Emeth, 1997; Plucker and Gorman, 1999; Backstrom and Bengtsson, 2019; Rangus and Cerne, 2019), our paper emphasizes on the career calling. We propose that career calling can motivate employees to innovate constantly, which is consistent with the values advocated by the society. Even if their innovation plans are rejected by leaders, they would continue to implement plans (Globocnik and Salomo, 2015; Lin et al., 2016). Therefore, through investigating the effect of career calling on employee creative deviance, this paper complements two streams of literature, i.e., employee creative deviance and career calling.

Second, our study validates the mediating effect of employee creativity. Existing studies on employee creativity primarily focus on leadership style instead of individual characteristics (Rego et al., 2014; Wang et al., 2019), hence our paper complements this field by focusing on the employee creativity. On the one hand, since employees with high-level career calling have stronger motivation for work achievement, they tend to increase work input and increase knowledge sharing with organizational members (Duffy et al., 2012a, 2013; Yao et al., 2020a), and thus generate more innovative ideas (Hirschi, 2012; Duffy et al., 2015). On the other hand, high-level career calling can inspire employees’ creativity, which can improve their critical thinking and divergent thinking to break the routine, question superiors and restructure knowledge (Hennessey and Amabile, 2010; Gino and Wiltermuth, 2014; Tan et al., 2017), thereby promoting creative deviance. Our findings offer novel empirical evidence on the mechanism between career calling and creative deviance, and complements research on the effect of creativity.

Finally, we explore a boundary condition in the relationship between career calling and creative deviance. Previous studies take individual’s job autonomy and thriving as boundary conditions (Jaiswal and Dhar, 2017; Jiang and Gu, 2017), while ignore situational factors such as leadership styles. In fact, leadership styles can exert a significant influence on employees’ behaviors (Velez and Neves, 2018; Badrinarayanan et al., 2019; Shakeel et al., 2020). We take ethical leadership as a moderator and find its moderating effect on the correlation career calling and employee creativity as well as the correlation between career calling and creative deviance, providing a new perspective for studying employee creativity and creative deviance.

### Practical implications

This study also bears implications in practice. First, leaders should realize the important role of employees’ career calling in organizational innovation. Since career calling can stimulate employees’ work enthusiasm and achievement motivation, the organization can enhance innovation ability by guiding employees to achieve self-worth and developing their career calling. Second, organizations should introduce employees’ creativity as an indicator of recruitment and promotion. Employees’ creativity directly affects their divergent thinking and critical thinking, which in turn can affect creative deviance. Therefore, the organization should consider the employee creativity in the recruitment and staff promotion to build a solid foundation for innovation. Third, leaders can adopt ethical leadership style, which can enhance the positive influence of career calling on employees’ creativity and creative deviance. They can care more about employees and make role modeling actively, thereby helping employees exhibit innovative behaviors.

### Limitations and future research directions

This study also subject to some limitations. First, although we collect questionnaires at two time points to reduce the common method variation, the career calling may vary with the change of external circumstances. Future studies can adopt longitudinal tracking surveys to get more accurate results and reduce the common method variation more effectively. Besides, although we have considered some demographic variables, there are still several factors at personal and occupational level have not been controlled, which may affect the reliability of results. Hence, we will control more personal and occupational level factors in subsequent studies. Second, this study collects data from single source. Following studies can conduct a matching survey between leaders and employees to make the data more objective. Third, the existing literature contends that mediating effects should consider both positive and negative aspects, such as emotional exhaustion and intrinsic motivation (Yao et al., 2020a,c). Future studies can examine other mediating mechanisms from both positive and negative sides. Fourth, in addition to the leadership style, some studies report that job stress may also affect personal traits’ impact on employee innovative behaviors (Yao et al., 2020b). Since time pressures and work bullying are common stress encountered by employees (Yao et al., 2020a), follow-up study can consider time pressure as the boundary condition.

## Data availability statement

The original contributions presented in the study are included in the article/supplementary material, further inquiries can be directed to the corresponding author.

## Ethics statement

Written informed consent was obtained from the individual(s) for the publication of any potentially identifiable images or data included in this article.

## Author contributions

XL conceived and designed the study, and completed the manuscript in English. XL and YX participated in drafting the manuscript and revised it critically for critical intellectual content. XL and YX gave much good research advice and revised the manuscript. YX improved the readability of the manuscript. All authors contributed to the article and approved the submitted version.

## Funding

This work was supported by the Hunan Province Education science “13th Five-Year Plan” project (XJK17BGD009) and the Research Foundation of the Hunan Provincial Education Department (20K035).

## Conflict of interest

The authors declare that the research was conducted in the absence of any commercial or financial relationships that could be construed as a potential conflict of interest.

## Publisher’s note

All claims expressed in this article are solely those of the authors and do not necessarily represent those of their affiliated organizations, or those of the publisher, the editors and the reviewers. Any product that may be evaluated in this article, or claim that may be made by its manufacturer, is not guaranteed or endorsed by the publisher.
